# Fatty Acyl Coenzyme A Synthetase Fat1p Regulates Vacuolar Structure and Stationary-Phase Lipophagy in Saccharomyces cerevisiae

**DOI:** 10.1128/spectrum.04625-22

**Published:** 2023-01-04

**Authors:** Fan Qiu, Na Kang, Jinling Tan, Sisi Yan, Leiying Lin, Lipeng Cai, Joel M. Goodman, Qiang Gao

**Affiliations:** a School of Biomedical Sciences, Hunan University, Changsha, Hunan, China; b Department of Pharmacology, University of Texas Southwestern Medical School, Dallas, Texas, USA; Chinese Academy of Sciences

**Keywords:** lipophagy, stationary phase, unsaturated fatty acid, vacuole microdomains, vacuole morphology

## Abstract

During yeast stationary phase, a single spherical vacuole (lysosome) is created by the fusion of several small ones. Moreover, the vacuolar membrane is reconstructed into two distinct microdomains. Little is known, however, about how cells maintain vacuolar shape or regulate their microdomains. Here, we show that Fat1p, a fatty acyl coenzyme A (acyl-CoA) synthetase and fatty acid transporter, and not the synthetases Faa1p and Faa4p, is essential for vacuolar shape preservation, the development of vacuolar microdomains, and cell survival in stationary phase of the yeast Saccharomyces cerevisiae. Furthermore, Fat1p negatively regulates general autophagy in both log- and stationary-phase cells. In contrast, Fat1p promotes lipophagy, as the absence of *FAT1* limits the entry of lipid droplets into the vacuole and reduces the degradation of liquid droplet (LD) surface proteins. Notably, supplementing with unsaturated fatty acids or overexpressing the desaturase Ole1p can reverse all aberrant phenotypes caused by *FAT1* deficiency. We propose that Fat1p regulates stationary phase vacuolar morphology, microdomain differentiation, general autophagy, and lipophagy by controlling the degree of fatty acid saturation in membrane lipids.

**IMPORTANCE** The ability to sense environmental changes and adjust the levels of cellular metabolism is critical for cell viability. Autophagy is a recycling process that makes the most of already-existing energy resources, and the vacuole/lysosome is the ultimate autophagic processing site in cells. Lipophagy is an autophagic process to select degrading lipid droplets. In yeast cells in stationary phase, vacuoles fuse and remodel their membranes to create a single spherical vacuole with two distinct membrane microdomains, which are required for yeast lipophagy. In this study, we discovered that Fat1p was capable of rapidly responding to changes in nutritional status and preserving cell survival by regulating membrane lipid saturation to maintain proper vacuolar morphology and the level of lipophagy in the yeast S. cerevisiae. Our findings shed light on how cells maintain vacuolar structure and promote the differentiation of vacuole surface microdomains for stationary-phase lipophagy.

## INTRODUCTION

Vacuoles in yeasts (homologous to lysosomes in animal cells) are highly dynamic organelles that undergo cycles of fission and fusion and can occupy as much as 10% to 20% of the cell volume depending on environmental conditions (1). In stationary phase (stat phase), vacuoles not only fuse but also remodel their membranes to form two distinct microdomains, a protein-enriched liquid-disordered (Ld) microdomain and a sterol- and sphingolipid-enriched liquid-ordered (Lo) microdomain (2–4). Along with preserving cellular homeostasis by storing ions, carbohydrates, amino acids, and phosphates, vacuoles are also the ultimate autophagic processing sites (5–7).

Autophagy is an evolutionarily conserved process in which vacuoles/lysosomes degrade various cellular components (8–10). Three types of autophagy are known, based on how cargo is delivered: macroautophagy, microautophagy, and chaperone-mediated autophagy (CMA) (11). In macroautophagy, a double-membrane structure, the phagophore, is formed around cargo, encapsulating it in a closed autophagosome in a matter of minutes (12, 13). Autophagosomes then fuse with vacuoles/lysosomes, releasing their cargo for degradation. In contrast, the vacuole/lysosome directly engulfs organelles or other cargo without the direct participation of autophagosomes in microautophagy (14, 15). Finally, selective proteins can be chaperoned to lysosomes via CMA (16, 17). Chaperones and specialized translocation complexes are required for CMA, a process which has not yet been identified in yeast (11).

Lipid droplets (LDs) are intracellular neutral lipid (NL) storage organelles found in all eukaryotic and many prokaryotic cells (18–20), and they provide cells with fatty acids (FAs) for β-oxidation and with lipids or lipid precursors for membrane expansion (21). Lipolysis and lipophagy are two principal processes for LD utilization (22–25). Lipolysis directly uses lipases to hydrolyze NLs in the cytosol, whereas lipophagy needs LDs to be delivered to the vacuole/lysosome for degradation. Lipophagy is a selective autophagic process that can be accomplished by both macroautophagy and microautophagy in mammals but only by microautophagy thus far in yeast (26). Although autophagosomes are not needed for yeast lipophagy, several core autophagy-related (ATG) genes, the endosomal sorting complex required for transport (ESCRT) complexes, and the Lo and Ld microdomains have been reported to be required (27, 28). Moreover, a bidirectional regulatory mechanism between lipophagy and vacuolar microdomain differentiation has also been proposed (29). Lipophagy, in this model, provides sterols to stimulate the formation of vacuolar microdomains, and then stabilized microdomains further secure and facilitate subsequent lipophagic events.

FATP family proteins are conserved from yeast to human, acting as both very-long-chain acyl coenzyme A (acyl-CoA) synthases (ACSs) and FA transmembrane transporters, with one member in Saccharomyces cerevisiae (Fat1p), two in Caenorhabditis elegans (ACS-20 and ACS-22), three in Drosophila melanogaster (Fatp, CG30194, and CG3394), and six in mammals (FATP1 to FATP6) (30, 31). Fatp1 is critical to the maintenance of retinal function in aged mice (32, 33). Elderly mice lacking *Fatp1* exhibit signs of age-related macular degeneration and thickening of the brush border layer, whereas younger mice do not. Studies also show correlations of FATP family proteins with plasma triglyceride levels, lipotoxic cardiomyopathy, FA uptake under cold conditions, and the size of LDs in brown adipose tissue (34–37). In yeast, Fat1p has also been reported to influence intracellular lipid saturation (38). Knockout of *FAT1* increases saturated lipids in cells, an effect that may be mediated by the fatty acyl desaturase Ole1p.

Our study began with an analysis of the role of acyl-CoA synthetases in autophagy under gradual starvation and rapamycin treatment conditions. Interestingly, we noticed that *FAT1* deletion elevated stat-phase autophagy but not rapamycin-induced autophagy. Fat1p is also important for the maintenance of stat-phase vacuolar morphology. Knockout of *FAT1* results in irregular nonspherical vacuoles and abnormal Lo and Ld microdomains, thereby suppressing lipophagy, as LDs must enter the vacuoles via Lo domains. Oleic acid incubation and Ole1p overexpression can rescue these deficiencies, suggesting that intracellular lipid saturation is dysregulated in *FAT1* mutant. Overall, our findings suggest that Fat1p regulates vacuolar morphology, membrane microdomain differentiation, and autophagic and lipophagic fluxes in stat-phase yeast cells by influencing the extent of intracellular lipid saturation.

## RESULTS

### Fat1p affects autophagic flux under gradual starvation.

A previous study reported that acyl-CoA synthetases (ACSs) Faa1p and Faa4p, but not Fat1p, are employed for autophagosome formation under nitrogen starvation and rapamycin treatment conditions (39). However, the mechanisms to initiate autophagy are demonstrated to be different under acute nitrogen deprivation, rapamycin treatment, and gradual starvation (40–44). The role of ACS proteins in regulating autophagy during progressive starving conditions has not been determined.

**FIG 1:**
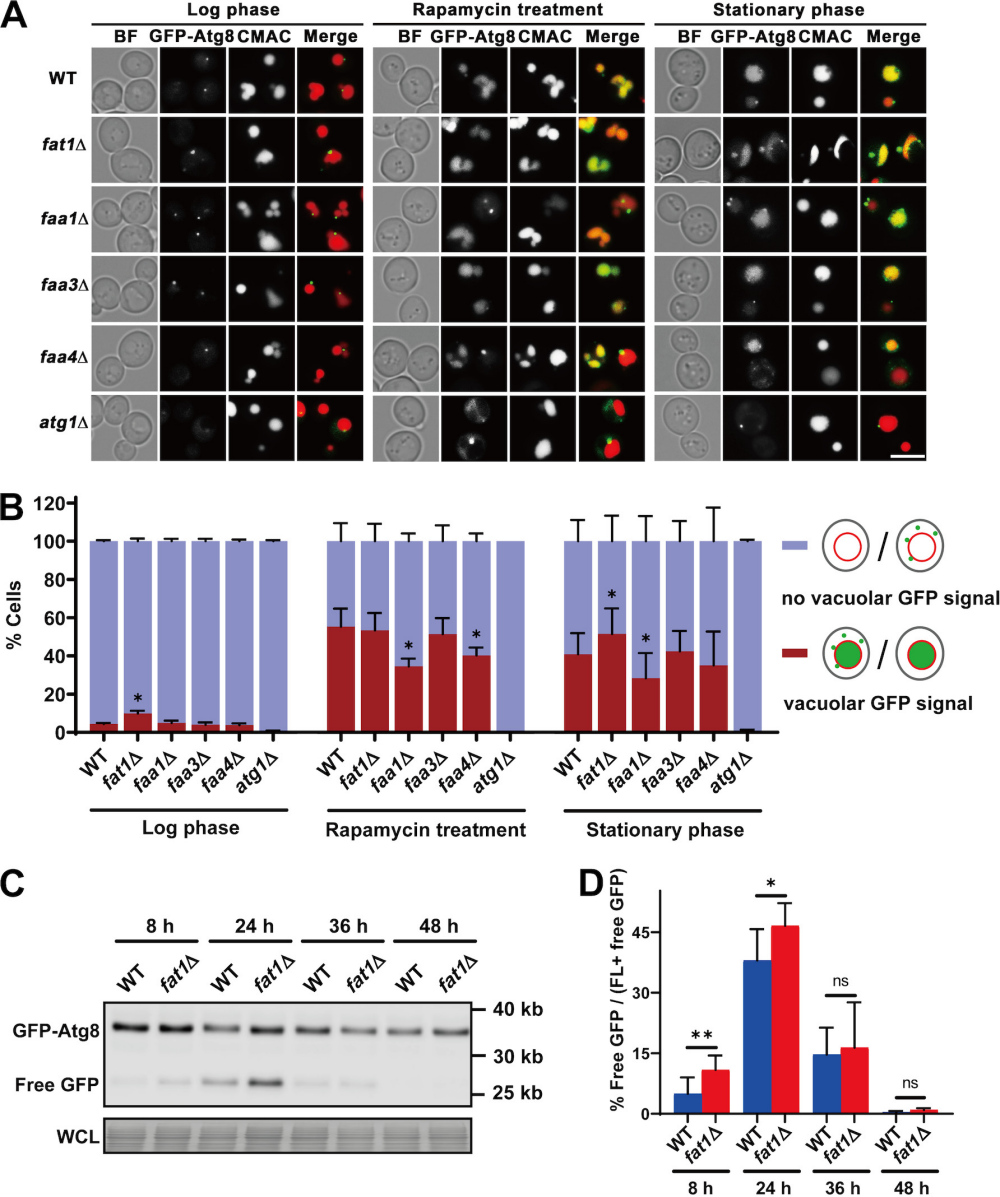
Fat1p restricts autophagic flux in the stat-phase yeast cells. (A) Cells from the indicated strains expressing chromosomal GFP-Atg8 were stained with CMAC to visualize vacuoles. Data from a representative experiment are shown. Left sections, log phase cells; middle sections, log phase cells treated with rapamycin for 3 h; and right sections, stat-phase cells. BF, bright field. Scale bar, 5 μm. (B) Quantification of cells from three replicate experiments with the indicated GFP-vacuole localization patterns in panel A. GFP signals were classified into two categories: cells with no vacuolar GFP signal and cells with intravacuolar GFP signal. (C) Wild-type (WT) and *FAT1* deletion (*fat1*Δ) strains containing GFP-Atg8 were grown in SD medium for the indicated hours. Cells were lysed, and the whole-cell lysates (WCL) were subjected to SDS-PAGE and immunoblotting with the anti-GFP antibody or staining with amido black. (D) Quantification of the degradation of GFP-Atg8. The data represent the free GFP/total GFP ratio in panel C. FL, full length. All quantitative data show means ± SD (*n *= 3 experiments). ns, no significance. *, *P < *0.05; **, *P < *0.01; ***, *P < *0.001, compared with WT.

In the yeast S. cerevisiae, general autophagic flux can be assessed by the localization and proteolytic release of green fluorescent protein (GFP) from GFP-labeled Atg8p (29, 45). We constructed *ACS* mutants expressing chromosomal GFP-Atg8 and cultured them in minimal synthetic dextrose (SD) medium to study the functions of these proteins in the autophagy process under gradual starvation and rapamycin treatment conditions (Fig. 1; see also Fig. S1 in the supplemental material). As a negative control, the *atg1*Δ mutant, which fails to form autophagosomes, containing GFP-Atg8 was also tested (46, 47). Autophagic flux was low in log-phase cells, as the wild type (WT) and most mutants only incorporated GFP-Atg8 into vacuoles in 5% or fewer cells; however, this was slightly higher in *fat1*Δ cells, at 10% of cells (Fig. 1A and B, left section; Fig. S1A). Autophagy, on the other hand, was rapidly promoted in all strains except the *atg1*Δ mutant (the negative control) when treated with rapamycin for 3 h or grown to stat phase (Fig. 1A and B, middle and right sections). Under these conditions, autophagosomes fused with vacuoles, and GFP-Atg8 degraded and released free GFP, resulting in GFP signals located in vacuoles. With rapamycin treatment, the percentages of cells with GFP-vacuole colocalization in the *faa1*Δ and *faa4*Δ mutants were lower than for the WT, while the *fat1*Δ and *faa3*Δ mutants showed no difference from the WT (Fig. 1A and B, middle section). These findings are consistent with the previous report that Faa1p and Faa4p are important for rapamycin-induced autophagy, whereas Fat1p and Faa3p are not (39). To our surprise, the *fat1*Δ mutant presented a higher ratio of cells with GFP-vacuole colocalization than the WT in stat phase, while the *faa1*Δ mutant had a lower ratio and the *faa3*Δ and *faa4*Δ mutants had no difference (Fig. 1A and B, right section). Immunoblotting analysis of GFP-Atg8 confirmed that stat-phase cells (24 h) had the highest level of autophagy (Fig. S1B and C). We compared the degradation of GFP-Atg8 of the WT and *fat1*Δ strains in SD at different growth periods (Fig. 1C and D). At 8 h and 24 h, the autophagic flux in the *FAT1* deletion strain was higher than that in the WT (Fig. 1C and D), which was consistent with the fluorescence imaging data (Fig. 1A and B). All of these results suggest that Fat1p negatively regulates general autophagy in both log and stat phases, while Faa1p positively regulates autophagy in stat phase and under the rapamycin treatment condition.

The number of GFP-Atg8 puncta in cells was also measured (Fig. S1D). Under stat phase and rapamycin treatment, the average number of GFP-Atg8 puncta in WT cells was slightly more than in log-phase cells (Fig. S1D). Mature autophagosomes are not actually formed in *ATG1* deletion strains (46, 47), but GFP-Atg8 puncta were still significantly increased in the *atg1*Δ strain (Fig. S1D). The number in the *atg1*Δ strain was comparable to those of the WT and other *ACS* mutants under both stat-phase and rapamycin treatment conditions, implying that calculating GFP-Atg8 puncta alone was insufficient to estimate autophagosome formation.

### Fat1p is involved in the maintenance of vacuolar morphology in stat-phase cells.

**FIG 2:**
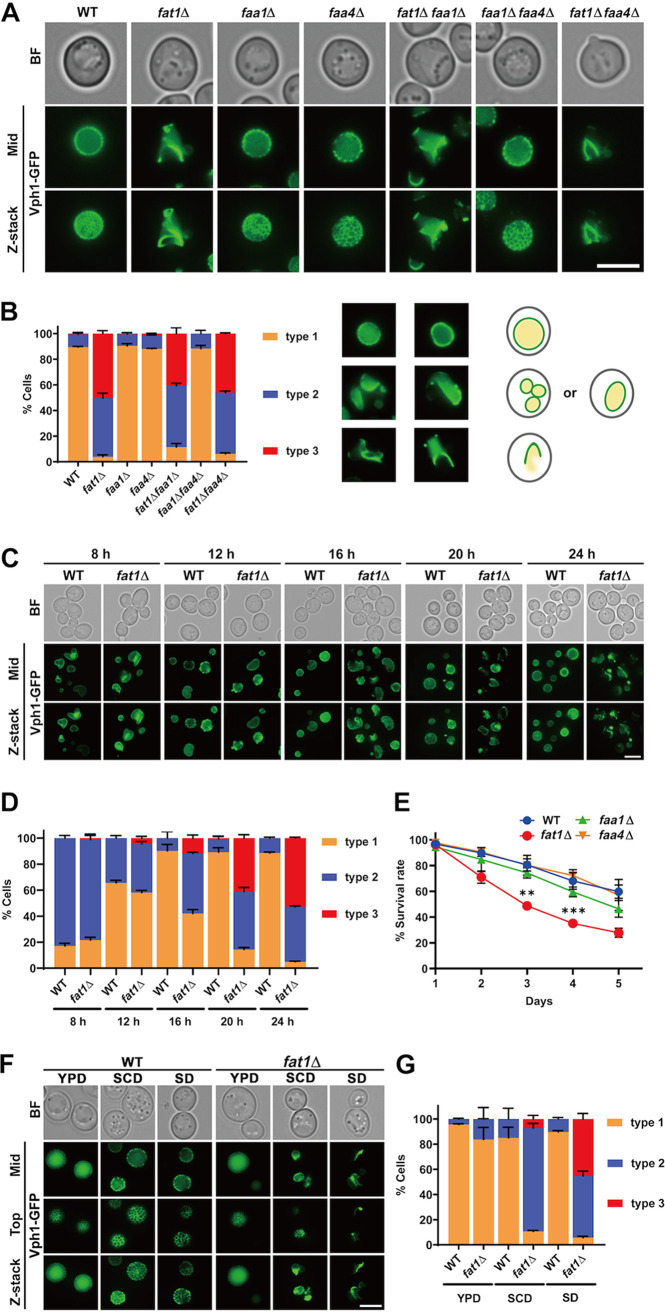
Fat1p is required for the proper stat-phase vacuole morphology. (A) Representative images of the indicated strains expressing chromosomal Vph1-GFP grown for 24 h to stat phase in SD medium. Cells with various types of vacuoles were scored as being in one of three categories: type 1, cells have a single spherical vacuole; type 2, cells have a single ellipsoidal vacuole or several small vacuoles; and type 3, cells have a nonspherical vacuole(s). (B) Quantification of cells with the indicated vacuole patterns in panel A. (C) WT and *fat1*Δ strains containing Vph1-GFP were cultured for the indicated hours. The log phase is represented by 8 h, the diauxic shift period is represented by 12 h and 16 h, and the early stat-phase is represented by 20 h and 24 h. (D) Quantification of the cells with the indicated vacuole patterns in panel C. (E) Survival curves of WT, *fat1*Δ, *faa1*Δ, and *faa4*Δ strains in SD medium. (F) Images of stat-phase WT and *fat1*Δ cells containing Vph1-GFP in YPD, SCD, and SD media. (G) Quantification of the number of cells with the indicated vacuole patterns in panel F. All data show means ± SD (*n *= 3). **, *P < *0.01; ***, *P < *0.001. Top, microscope focus on top tangential area of the vacuole; mid, focus on the midsection of the vacuole; Z-stack, maximal superimposed projection of vacuole images. Scale bar, 5 μm.

Typically, yeast cells have several irregularly shaped vacuoles in log phase. As cells reach the diauxic shift phase, these vacuoles fuse to form a single round vacuole, and this spherical morphology is maintained throughout the subsequent stat phase (48). In the *fat1*Δ mutant, vacuoles displayed a variety of aberrant, nonspherical shapes in SD during stat phase (Fig. 1A). We labeled the vacuolar membranes in the WT and *ACS* mutant strains with chromosomal GFP-tagged Vph1p (Fig. 2; Fig. S2A to C). Vacuoles of all strains showed no detectable differences in log phase (Fig. S2A), whereas vacuoles in *fat1*Δ cells were ellipsoid or other irregular shapes in stat phase compared to normal spherical vacuoles in the WT, *faa1*Δ, and *faa4*Δ strains (Fig. 2A and B; Fig. S2B). This vacuole defect was also found in *fat1*Δ *faa1*Δ and *fat1*Δ *faa4*Δ strains but not in the *faa1*Δ *faa4*Δ strain (Fig. 2A and B). We also found that this aberrant vacuolar phenotype first appeared in the *fat1*Δ mutant at the diauxic shift period (Fig. S1A) and persisted throughout the whole stat phase (Fig. 2C and D).

As morphological defects of the vacuoles may result in a decrease in cell viability (49, 50), we asked whether deletion of *FAT1* affected cell survival. Indeed, the survival rate of the *fat1*Δ mutant in SD medium was significantly lower than those of the WT, *faa1*Δ, and *faa4*Δ strains at 72 h (Fig. 2E), and this difference was maintained at least through 4 days. These findings indicate that Fat1p, and not Faa1p or Faa4p, plays a distinct role in maintaining vacuolar structure and cell survival during the nutrient-depleted stat-phase period.

This Fat1p-associated vacuolar defect was completely dependent on nutritional conditions (Fig. 2F and G). Three culture media were evaluated: yeast extract-peptone-dextrose (YPD), complete synthetic dextrose (SCD), and SD. YPD is the most nutritionally rich medium, while SD is the least. In log phase, the vacuoles of WT and *fat1*Δ cells showed no differences in all of these media (Fig. S2C). In contrast, while WT cells had consistent globular vacuoles in all media, *fat1*Δ cells had vacuoles of remarkably different morphologies in stat phase, depending on the medium (Fig. 2F and G). Vacuoles of *fat1*Δ cells, which had high proportion of spherical shapes in YPD, became largely ellipsoid in SCD and even more irregular in SD (Fig. 2F and G). These findings indicate that culture conditions greatly influence the stat-phase vacuolar structure of *FAT1* deletion cells.

### Fat1p is crucial for stat-phase lipophagy.

Cellular localization of Fat1p, Faa1p, and Faa4p was examined in SD medium (Fig. S2D to F). Faa1p was found in the endoplasmic reticulum (ER), as previously reported, while Fat1p and Faa4p were highly enriched on LDs (51–53). The localization of Fat1p on LDs, effects on autophagic flux, vacuolar morphology, and cell survival all point to Fat1p being involved in regulating lipophagy, a degradation process in which LDs are directly (in yeast) internalized by vacuoles and hydrolyzed to release FAs during stat phase (25).

**FIG 3:**
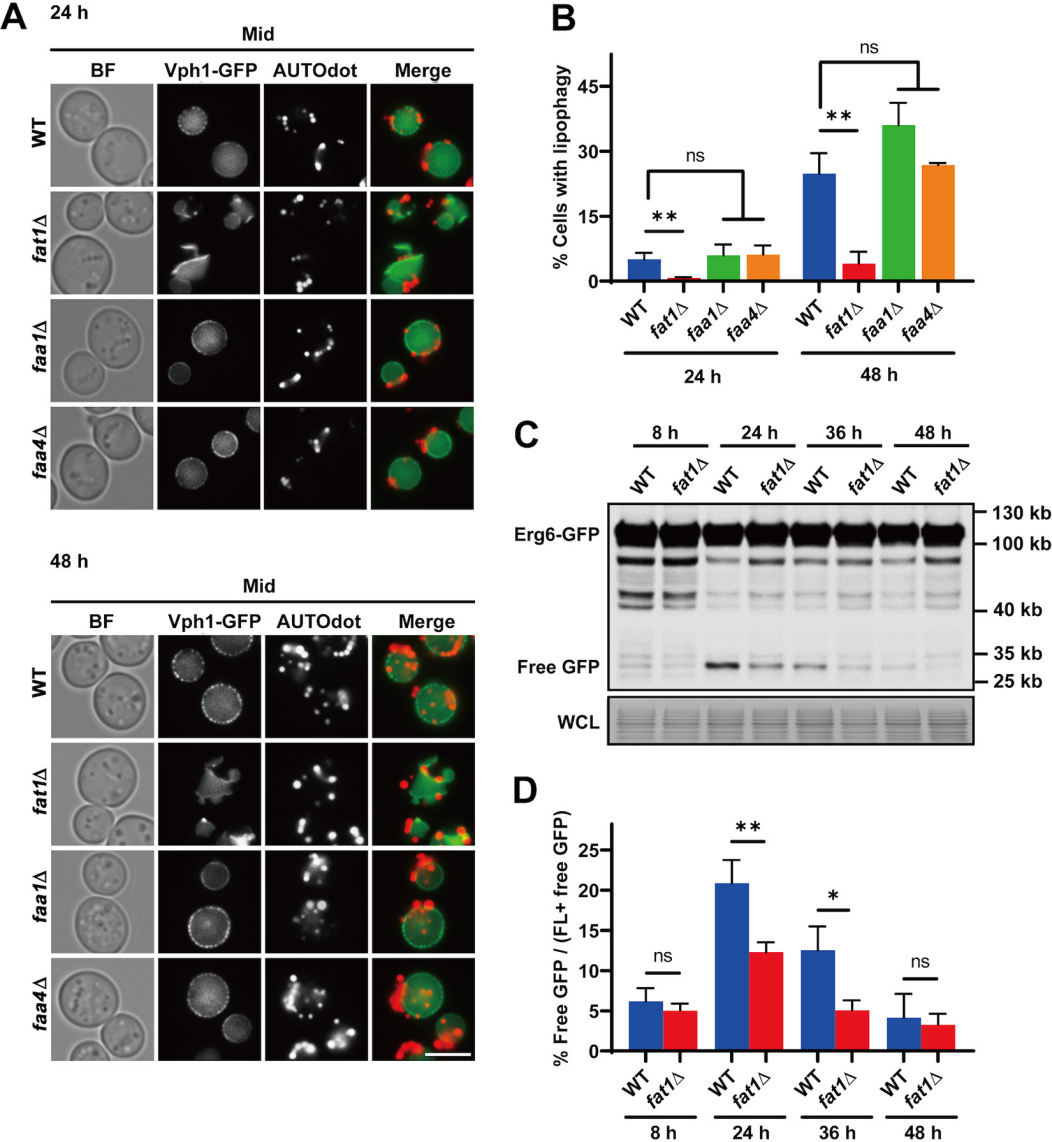
Fat1p is required for stat-phase lipophagy. (A) Lipophagy was assessed in cells of the indicated strains containing Vph1-GFP using AUTOdot dye in stat phase. Lipophagy was defined as LDs engulfed in vacuoles. Scale bar, 5 μm. (B) Quantification of stat-phase lipophagy in panel A. (C and D) Whole-cell lysates from the indicated strains were immunoblotted with anti-GFP antibody or stained with amido black to assess the degradation of Erg6-GFP. The free GFP/total GFP ratios were calculated. All data show means ± SD (*n *= 3). *, *P < *0.05; **, *P < *0.01.

Lipophagy can be measured either directly by observing the localization of LDs with vacuoles or indirectly by monitoring the degradation of LD surface proteins. AUTOdot dye was used to visualize LDs in SD medium in wild-type, *fat1*Δ, *faa1*Δ, and *faa4*Δ cells containing Vph1-GFP. Lipophagy was first observed in SD at 24 h (Fig. 3A). At this time, LDs clustered on the vacuolar surface and had begun to gradually invaginate into the vacuolar lumen. Approximately 5% of wild-type, 6% of *faa1*Δ, and 6% of *faa4*Δ cells had LDs engulfed in vacuoles and undergoing lipophagy, but this rate was less than 1% in *fat1*Δ cells (Fig. 3B). At 48 h, cells displaying lipophagy increased to 25% of wild type, 36% of *faa1*Δ cells (not significantly different from the WT; *P* = 0.11), and 27% of *faa4*Δ cells, while only 4% of *fat1*Δ cells were undergoing lipophagy and almost all LDs remained on the vacuolar surface (Fig. 3A and B). We also created wild-type and *fat1*Δ strains expressing chromosomal Erg6-GFP to estimate the degradation of LD surface proteins. Immunoblotting analysis revealed that the levels of autophagic degradation of Erg6p in *fat1*Δ were also lower than those in the wild type at 24 h and 36 h (Fig. 3C and D). These morphologic and biochemical results both suggest that Fat1p plays an important role in lipophagy in stat-phase cells.

### Fat1p influences the formation of stat-phase vacuolar microdomains.

**FIG 4:**
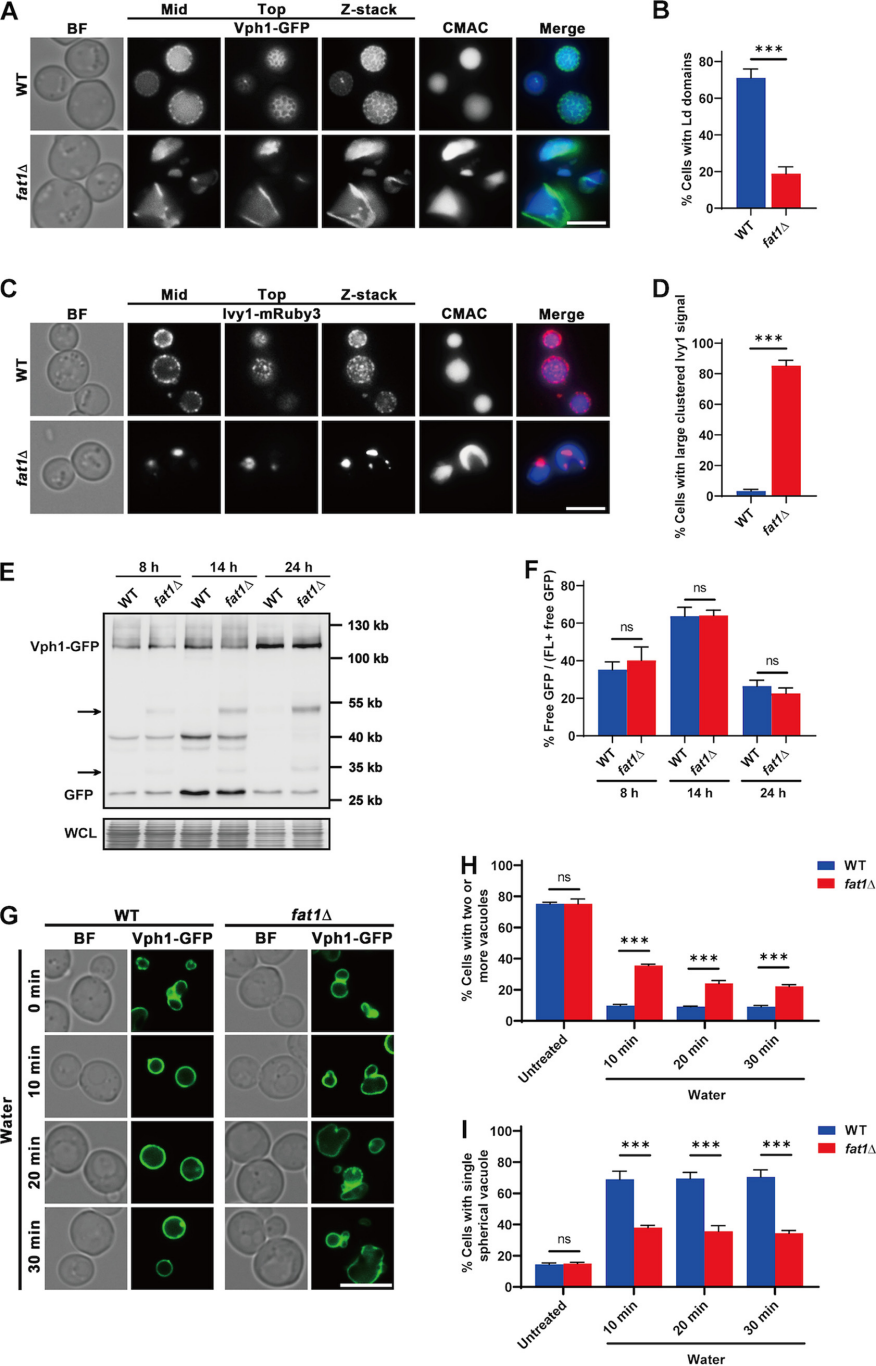
Fat1p influences vacuolar microdomain formation. (A) GFP-tagged Vph1 of WT and *FAT1* deletion cells were imaged after CMAC staining to show the liquid-disordered (Ld) microdomains. (B) Quantification of data shown in panel A. (C) WT and *fat1*Δ cells expressing Ivy1-mRuby3 were imaged with CMAC dye to reflect the liquid-ordered (Lo) microdomains. (D) Quantification of data shown in panel C. (E) Whole-cell lysates from the indicated strains were immunoblotted with anti-GFP antibody or stained with amido black to assess the degradation of Vph1-GFP at different growth phases. The arrows indicate partially degraded Vph1-GFP bands. (F) Quantification of the degradation of Vph1-GFP in panel E. The free GFP/total GFP ratios are shown. (G) Vacuolar morphology of indicated strains after hypoosmotic shock. Cells were grown in SD medium to log phase before being resuspended in water and imaged at the indicated times. (H and I) Quantification of data presented in panel G. (H) Percentage of cells containing two or more vacuoles at the indicated times. (I) Percentage of cells containing one single spherical vacuole at the indicated times. All data show means ± SD (*n *= 3). ***, *P < *0.001. Top, microscope focus on top tangential area of the vacuole; mid, focus on the midsection of the vacuole; Z-stack, maximal superimposed projection of vacuole images. Scale bar, 5 μm.

We think that Fat1p is involved in the formation of stat-phase vacuolar microdomains due to abnormal vacuolar morphology and blocked lipophagic flux. Vph1p has proven to be selectively distributed in the Ld microdomains, while Ivy1p is found exclusively in the Lo microdomains (4, 29). In stat phase, Ld microdomains displayed a mesh polygonal network in 71% of WT and 19% of *fat1*Δ cells, whereas the distribution of Vph1-GFP shifted to a stripe-like structure in most *fat1*Δ cells (Fig. 4A and B; Movies S1 and S2). Correspondingly, 97% of WT cells had a relatively uniform punctate distribution of Lo microdomains, while 84% of *fat1*Δ cells had only one or a few aggregated clusters of Ivy1-mRuby3 (Fig. 4C and D; Movies S3 and S4). The expression and degradation of Vph1-GFP in WT and *fat1*Δ cells exhibited similar patterns with minor differences (Fig. 4E and F), although the samples of *fat1*Δ cells had two additional incomplete degradation bands compared to the WT (Fig. 4E, arrows), indicating that the degradation of Vph1-GFP in *fat1*Δ cells was partially impaired. In summary, our findings support the notion that Fat1p regulates the formation of dispersed Lo and Ld microdomains in stat phase.

The vacuole, which is rich in various ions and a variety of hydrolytic enzymes, is important for nutrient sensing and homeostatic regulation in yeast, as well as being the ultimate site for degrading many proteins (5). It needs to maintain an internal acidic environment to ensure that protein hydrolases function properly (7). We evaluated several basic physiological properties of the vacuole in WT and *FAT1* mutant cells, including vacuolar acidity, protein sorting properties to the vacuole, and tolerance to acidic and alkaline pHs and hyper- and hypoosmotic conditions (Fig. S3). We only found a difference when cells were subjected to hypotonic treatment by addition of water during log phase (Fig. 4G to I). Vacuolar dynamics, however, were considerably altered in *fat1*Δ cells. Small vacuoles in wild-type cells can fuse quickly to form a single large spherical vacuole in minutes (54, 55). In *fat1*Δ cells, vacuole fusion was delayed, and the shape of the fused vacuole was abnormal (Fig. 4G to I). A total of 35% of *fat1*Δ cells had two or more vacuoles after being cultured in water for 10 min, whereas only 10% of WT cells had more than one vacuole (Fig. 4G and H). Moreover, at 10 min, 69% of WT cells had a typical spherical vacuole, while only 38% of *fat1*Δ vacuoles had this shape, which did not improve even after 30 min of treatment (Fig. 4G and I).

### Fat1p regulates the membrane lipid saturation in stat phase.

The physicochemical properties of the cell membrane, such as stiffness, phase behavior, and fluidity, are strongly influenced by the composition and saturation level of lipids (56). Cells can regulate surface properties of their membranes and identity of organelles and maintain functional secretory pathways by adjusting lipid saturation (57, 58). Furthermore, increased lipid saturation could cause ER stress and activate the unfolded protein response (UPR) (59, 60). In some extreme cases, changes in membrane lipid saturation can cause large alterations in organellar abundance and morphology and even cell death (57, 60–62).

**FIG 5:**
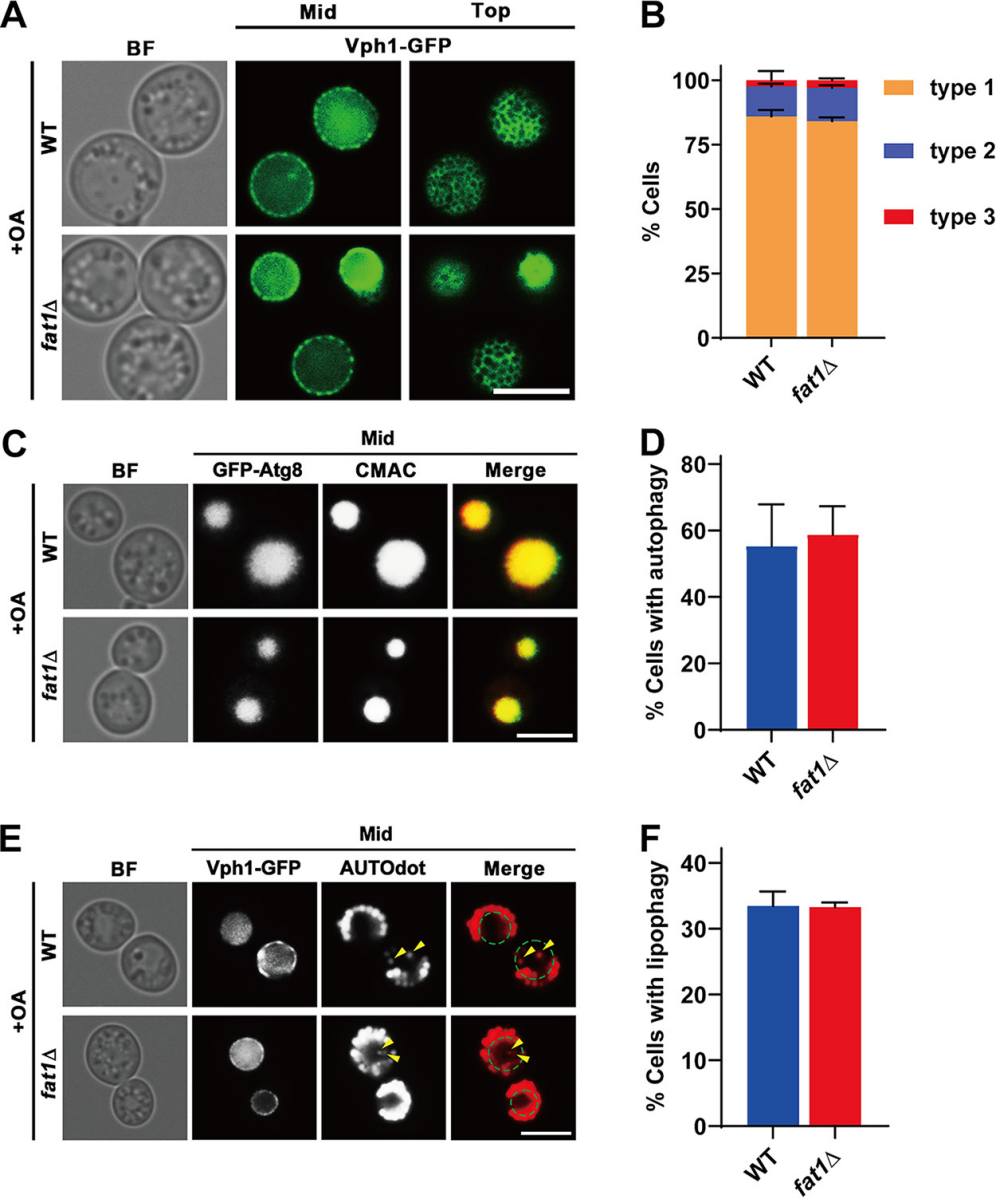
Exogenous oleic acid supplementation reverses vacuole defects in *fat1*Δ strains. (A) Wild-type (WT) and *FAT1* deletion (*fat1*Δ) strains containing Vph1-GFP were cultured in OA medium for 24 h. See Fig. 2 legend for description of vacuolar types. (B) Quantification of data shown in panel A. (C) Fluorescence imaging of indicated strains cultivated in OA medium for 24 h. To assess autophagy, cells from the indicated strains containing GFP-Atg8 were stained with CMAC dye. (D) Quantification of data shown in panel C. (E) Cells of the indicated strains containing Vph1-GFP were grown in OA for 48 h and then stained with AUTOdot dye to evaluate lipophagy. Yellow arrowheads indicate LDs in the vacuole. (F) Quantification of data shown in panel E. All data show means ± SD (*n *= 3). Top, microscope focus on top tangential area of the vacuole; mid, focus on the midsection of the vacuole. Scale bar, 5 μm.

An earlier study reported that fatty acyl groups in phosphatidylcholine (PC) were more saturated in *fat1*Δ cells (38). We suspected that the abnormal morphology and functional defects of the vacuole in the *FAT1*-deficient strain were attributable to lipid saturation dysregulation. As exogenous oleic acid (OA) can remarkably increase intracellular unsaturated lipids (63), we performed an OA culture assay to test whether supplementing with unsaturated FAs could reverse the phenotypes caused by *FAT1* deletion. After 24 h of culturing cells in OA medium, the distribution of Ld microdomains and vacuolar morphology of the *fat1*Δ strain were efficiently restored to that of the WT (Fig. 5A and B). Meanwhile, OA supplementation restored both autophagic and lipophagic defects in *FAT1*-deficient cells (Fig. 5C to F). These findings suggest that the addition of unsaturated FAs is able to reverse a wide range of abnormal phenotypes caused by *FAT1* deficiency.

**FIG 6:**
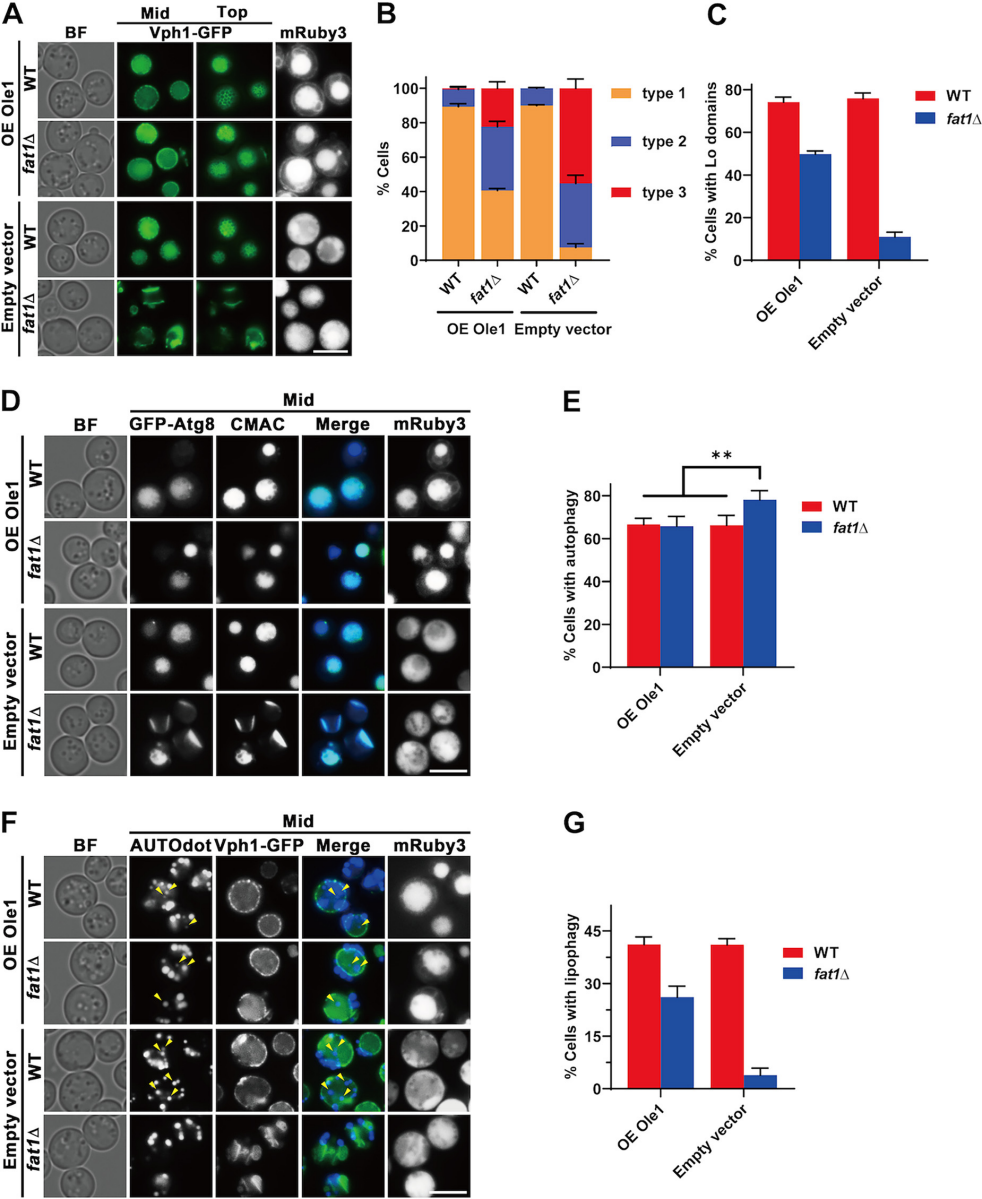
Fat1p regulates vacuolar membrane lipid saturation in stat-phase cells. (A to G) Effects of Ole1p overexpression on *FAT1* deletion strains. Ole1-mRuby3 or mRuby3 was overexpressed in a plasmid containing the strong *PGK1* promoter. (A) WT and *FAT1* deletion (*fat1*Δ) cells overexpressing Ole1-mRuby3 (OE Ole1) or mRuby3 (empty vector) were cultured in OA medium for 24 h. See Fig. 2 legend for description of vacuolar types. (B and C) Quantification of data shown in panel A. (D) WT and *FAT1* deletion (*fat1*Δ) GFP-Atg8 cells overexpressing either Ole1-mRuby3 or mRuby3 alone were cultured in OA medium for 24 h and then stained with CMAC dye to evaluate autophagy. (E) Quantification of autophagy shown in panel D. (F) WT and *FAT1* deletion (*fat1*Δ) Vph1-GFP cells overexpressing Ole1-mRuby3 or mRuby3 were cultured in OA medium for 48 h and then stained with AUTOdot dye to evaluate lipophagy. (G) Quantification of lipophagy shown in panel F. All data are means ± SD (*n *= 3). **, *P < *0.01. Top, microscope focus on top tangential area of the vacuole; mid, focus on the midsection of the vacuole image. Scale bar, 5 μm.

Ole1p is the only fatty acyl desaturase found in S. cerevisiae (64). Deletion of *FAT1* had no effect on *OLE1* mRNA or Ole1p expression levels, but it did result in a lower Ole1p activity (63). If this were true (63), then Ole1p overexpression may compensate, at least partially, for the loss of *FAT1*. To test this, Ole1-mRuby3 were overexpressed in both wild-type and *fat1*Δ strains. As predicted, overexpression of Ole1p partially restored vacuole morphology and Lo microdomains (Fig. 6A to C). When Ole1-mRuby3 was overexpressed in the *fat1*Δ strain, the rate of cells with normal spherical vacuoles increased from 8% to 41%, although it was still lower than for the WT (90%) and WT-overexpressed Ole1-mRuby3 (89%) (Fig. 6B). Cells with normal Lo microdomain distribution in *fat1*Δ strain-overexpressed Ole1-mRuby3 increased from 11% to 50%, while WT and WT-overexpressed Ole1-mRuby3 were 76% and 74%, respectively (Fig. 6C). Autophagic flux was restored to WT levels in *fat1*Δ strain-overexpressed Ole1-mRuby3 (Fig. 6D and E), while lipophagy was restored from 4% to 26% (Fig. 6F and G). In conclusion, Fat1p may regulate membrane lipid saturation by influencing the activities of Ole1p, though the mechanism requires further investigation.

## DISCUSSION

We report herein that Fat1p, a LD-localized ACS protein, plays an important role in maintaining vacuolar morphology and regulating autophagy and lipophagy in stat phase, by regulating FA saturation of lipids. Compared to the case with wild-type cells, *FAT1* deletion results in irregular nonspherical vacuole shapes, abnormal distribution of liquid-ordered and liquid-disordered microdomains, and increased autophagy and decreased lipophagy.

Fat1p plays a unique role in vacuolar morphology maintenance in stat-phase cells (Fig. 2 and 4). The viewpoint is supported by a number of pieces of evidence. First, stat-phase *fat1*Δ cells have irregular nonspherical vacuoles, which distinguishes them from vacuolar defects caused by increased fission, impaired fusion, or failure in the vacuolar targeting protein sorting pathway (50, 65). Second, this phenotype exists only in strains with *FAT1* deletion, whereas vacuoles are normal in *faa1*Δ, *faa4*Δ, and *faa1*Δ *faa4*Δ cells. This suggests that Fat1p is the only ACS protein involved in regulating vacuole morphology. Third, Fat1p has no effect on the inheritance or fusion process of vacuoles. In stat phase or under hypotonic conditions, vacuoles in *fat1*Δ cells could still fuse into a single one. Fourth, Fat1p influences vacuole microdomain differentiation, but this depends on the initial nutritional environments. A sophisticated signaling transduction network regulates the physiological and metabolic status of the cell in response to environmental nutritional conditions; even minor changes can trigger completely different signaling or metabolic regulation processes (66, 67). More study is needed to determine the mechanism by which deletion of *FAT1* affects the morphology and function of vacuoles only under nutritionally limited conditions.

According to a recent study, phagophores recruit ACS family members Faa1p and Faa4p, but not Fat1p, *in situ* to activate free FAs into phospholipid synthesis and drive autophagic membrane expansion under nitrogen starvation or rapamycin treatment conditions (39). We found that Fat1p is indeed not involved in the regulation of autophagy under rapamycin treatment, as previously reported, but deletion of *FAT1* leads to increased autophagy under gradual starvation, whereas the *FAA1* mutant results in decreased autophagy under both conditions (Fig. 1). These results suggest that Fat1p regulates stat-phase autophagy through a mechanism distinct from that of Faa1p and Faa4p in autophagosome membrane extension. In *FAT1* mutants, the formation and differentiation of Lo and Ld microdomains on vacuoles are also abnormal; as a result, LDs fail to enter the vacuoles for degradation (Fig. 3 and 4). All of these findings imply that lipophagy is also affected in the mutants. It seems contradictory that knockout of *FAT1* reduces lipophagy while promoting autophagy, but the two are not incompatible. In yeast, the mechanisms of autophagy and lipophagy are not the same. The formation of autophagosomes is required for autophagy but not for lipophagy in yeast (25, 29, 68), while the differentiation of vacuolar microdomains is essential for stat-phase lipophagy but not for autophagy (27, 29, 69). Fat1p may influence autophagy via modulating lipophagy. As cells are defective in lipophagy in *FAT1* deletion strains, FAs released from neutral lipids are insufficient for subsequent oxidative energy supply to maintain survival when environmental nutrition is depleted; as an alternative, cells enhance autophagic degradation of other nonessential components to produce energy. Thus, in the *FAT1* deletion strain, increased autophagy and decreased lipophagy can coexist without being contradictory.

Fat1p regulates FA homeostasis. It is a very-long-chain acyl-CoA synthase that participates in FA transmembrane transport (70–73) and has been shown to affect FA saturation of phospholipids (38). We think that the regulatory effects on vacuole morphology, as well as on associated autophagy and lipophagy, are related to the function of Fat1p in FA saturation control. Several pieces of evidence can back up our assumption. First and foremost, there are no FAs in the SD medium. As a result, the function of FA transport across the plasma membrane can be ignored or regarded as a minor component. Second, vacuole-associated defects in the *fat1*Δ strain can be rescued when the strain is cultured with OA (Fig. 5). Oleic acid is a C_18_ monounsaturated FA, whereas the core catalytic substrates of Fat1p are C_20_ to C_26_ FAs (71, 72). Third, overexpression of Ole1p, the only desaturase in S. cerevisiae, can partially restore most of the defects caused by *FAT1* deficiency (Fig. 6). All of these findings support our assumption that Fat1p regulates vacuole morphology and associated autophagy and lipophagy by facilitating the lipid saturation of the vacuole membrane.

**FIG 7:**
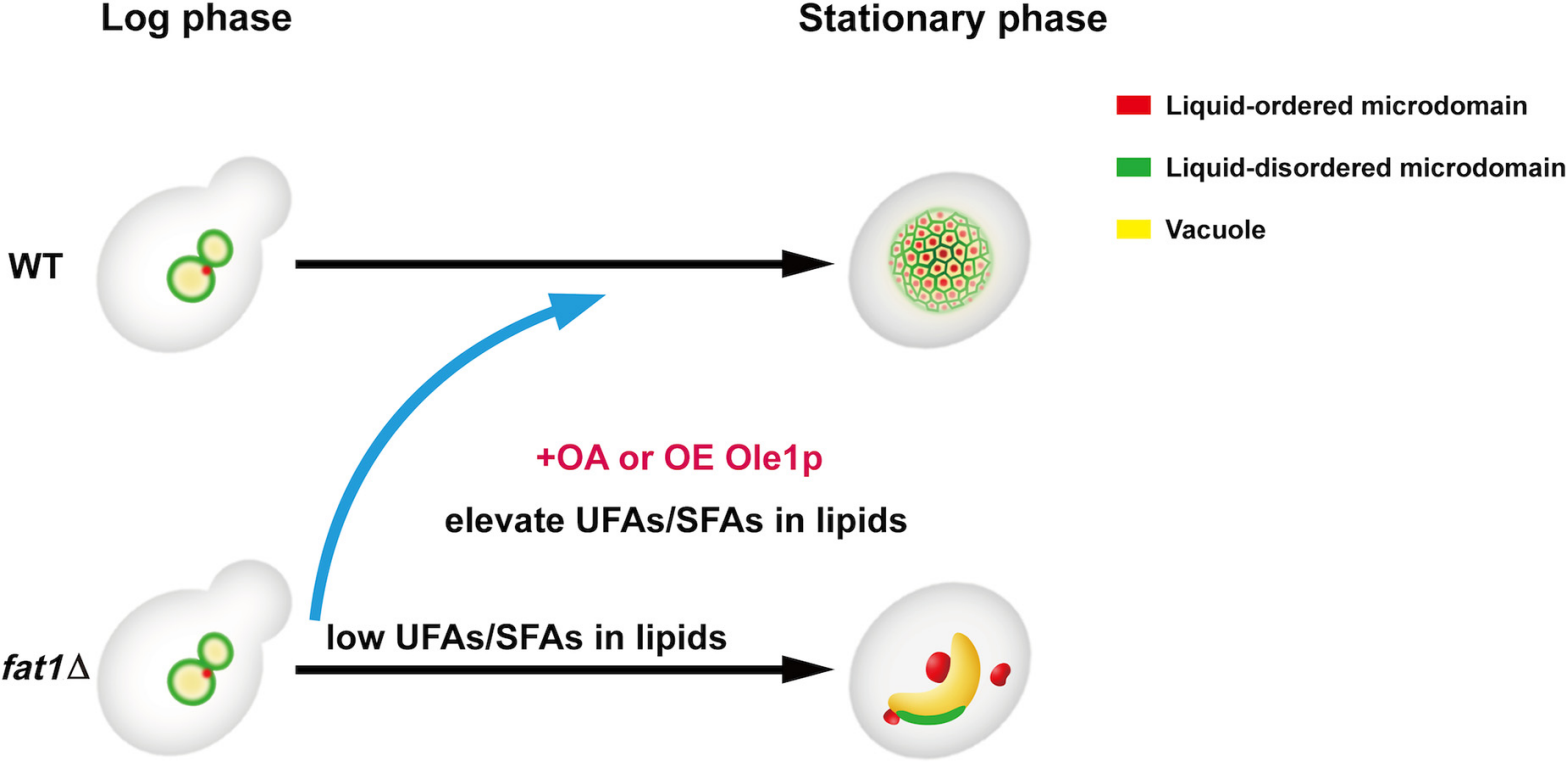
Model of Fat1p regulating stat-phase vacuole structure and lipophagy. Yeast cells typically have more than one vacuole in log phase. When cells reach the stat phase in SD medium, these vacuoles fuse into a spherical one with a typical mesh polygonal distribution of microdomains. Although vacuoles in *FAT1* mutants can fuse, their shapes change from spherical to irregular, and the normal distribution of Lo and Ld microdomains fails. This phenotype can be restored by supplementing with OA or overexpressing Ole1p. The low unsaturated FA/saturated FA ratio in lipids shown in *FAT1* deletion mutants is based on previously published work (38). UFAs, unsaturated FAs; SFAs, saturated FAs.

Based on our findings, we propose that Fat1p regulates vacuole morphology and function as the following model (Fig. 7). By regulating FA saturation of membrane lipids, yeast cells adjust the fluidity and rigidity of the vacuole. When environmental nutrients become scarce, cells rebuild vacuoles to create a single spherical vacuole with specialized microdomains for and lipophagy. Fat1p deletion disrupts the regulation of vacuole lipid saturation, resulting in a nonspherical vacuole with an abnormal distribution of liquid-ordered and liquid-disordered microdomains. Failure to form proper microdomains results in a blockage of lipophagy, exacerbating the defects of the vacuole. Lipophagy flaws also result in a failure to provide sufficient FAs for energy production, which indirectly contributes to the elevated level of autophagy required for survival.

## MATERIALS AND METHODS

### Strains, plasmids, and reagents.

The strains used in this study are listed in Table S1 and are all based on W303-1A (74). For increasing unsaturated lipids in cells, Ole1p was overexpressed by inserting the *OLE1* coding sequence with a C-terminal mRuby3 between the *PGK1* promoter and *PGK3* terminator to create pRS316-PGK1-Ole1p-mRuby3 using a modified pRS316 plasmid (75).

The media used in this study are listed in Table S2. Difco yeast nitrogen base, yeast extract, Bacto peptone, Bacto agar, and Difco LB broth were all BD products. BODIPY 493/503 and CMAC (7-amino-4-chloromethylcoumarin) dye were purchased from Invitrogen, whereas rapamycin and propidium iodide were acquired from MACKLIN. AUTOdot was purchased from abcepta, and oleic acid was from Sangon Biotech. All other reagents were obtained from Sigma-Aldrich. Anti-CPY (carboxypeptidase Y) (Saccharomyces cerevisiae) and anti-GFP antibodies were acquired from Abcam and Proteintech, respectively.

### Growth conditions.

All strains were cultured at 30°C on minimal synthetic dextrose (SD) medium at 210 rpm, unless otherwise stated. Before being diluted to 0.1 OD_600_ (optical density at 600 nm) unit/mL and cultivated for 24 h, cells were precultured for an overnight period at 30°C and 210 rpm. Cells were then diluted once more in SD to 0.1 OD_600_ unit/mL and cultivated for 1 to 3 days for the following tests. To induce autophagy, 400 ng/mL rapamycin was added to the medium when cells were incubated in SD for 8 h (39). For oleate cultures, a modified version of a previous procedure (39, 76, 77) was used; cells were precultured overnight, diluted to 0.1 OD_600_ unit/mL in SD containing 0.2% glucose, incubated for 12 h, washed, resuspended to 0.5 OD_600_ unit/mL in OA medium, and grown for 24 h.

### Fluorescence microscopy.

Microscopic was performed as described previously (78), except that the acquiring microscope was an Olympus-BX63 and the camera was from Teledyne Photometrics (model 01-PRIME-BSI-R-M-16-C). The objective used was a 100× 1.45 numerical aperture (NA) universal superflat field super compound achromatic oil immersion lens (Olympus; UPLXAPO100XO). An infinity-corrected optical system was used, with a flush focus distance of 45 mm at international standards, motorized focusing, and a focus accuracy of 10 nm. Image analysis was performed using the image analysis software cellSens Dimension (CS-DI-V3). The filters used with the cellSens Dimension system were 510/50 nm for GFP and quinacrine dye, 575/25 nm for mRuby3, and 420/60 nm for CMAC and propidium iodide (PI) stain. For lipid droplet image acquisition, living cells were stained with BODIPY (1:1,000 dilution of a 1-mg/mL stock in dimethyl sulfoxide [DMSO]) for 20 min or AUTOdot (1:500 dilution of a 0.1 M stock in DMSO) for 30 min. To image vacuoles, living cells were incubated in CMAC (25 μg/μL) dye solution for 30 min protected from light. For PI staining imaging, cells were stained with propidium iodide (1:200 dilution of a 1-mg/mL stock in double-distilled water [ddH_2_O]) for 5 min. For measurement of vacuolar pH, living cells were stained with 400 μL quinacrine dye (containing 50 mM Na_2_HPO_4_ [pH 7.6] and 0.2 mM quinacrine dihydrochloride) for 40 min at room temperature, bathed in ice for 15 min, and then washed and resuspended with buffer solution (100 mM HEPES, 50 mM Na_2_HPO_4_ [pH 7.6], 2% glucose) 3 times. A Z-stack of 20 to 25 images spaced 0.3 μm apart were collected with cellSens Dimension, and the maximum projections were generated using nearest-neighbor deconvolution. Microscope intensity settings and acquisition times were the same for all samples in an experiment unless otherwise stated in a figure legend.

### Immunoblotting.

At the indicated time points, 30 OD_600_ units of cells were harvested. All of these cells were used to prepare whole-cell extracts with 10% trichloroacetic acid, which were then resuspended in 300 μL bromophenol-free 2× Laemmli sample buffer and neutralized to pH 6 to 9. Cells were shattered by vortexing with 0.3 g acid-washed glass beads. The samples were boiled for 10 min and then centrifuged at 13,000 rpm for 5 min. Comparable samples (0.5 to 1.0 OD_600_ unit) were loaded onto SDS-PAGE gels and immunoblotted with ECL. The signal acquisition, processing, and quantification were performed by use of an imager (OI600; BIO-OI) and ImageJ software.

### Replications and statistical analyses.

All experiments were repeated at least twice (i.e., three independent biological replicates), with similar results. Micrographs depict one of these experiments. All repetitions were counted in the quantified data. Unless stated otherwise, a two-tailed, unpaired Student *t* test was used to assess differences between two groups. Analysis of variance (ANOVA) was used to compare multiple samples, and then Tukey’s test was used to compare pairs of samples. GraphPad Prism 8 software was used to create and analyze all statistical plots. The error bars are always displayed as ±standard deviations (SD).
