# Supplementary material for: Fatty Acyl Coenzyme A Synthetase Fat1p Regulates Vacuolar Structure and Stationary-Phase Lipophagy in Saccharomyces cerevisiae
Source: Microbiol Spectr. 2023 Jan 4;11(1):e04625-22. doi: 10.1128/spectrum.04625-22 (PMC9927365; doi:10.1128/spectrum.04625-22)
Supplement: Supplemental file 1 — Fig. S1 to S3, Tables S1 and S2, and legends of Movies S1 to S4. Download spectrum.04625-22-s0001.pdf, PDF file, 0.6 MB [file spectrum.04625-22-s0001.pdf]

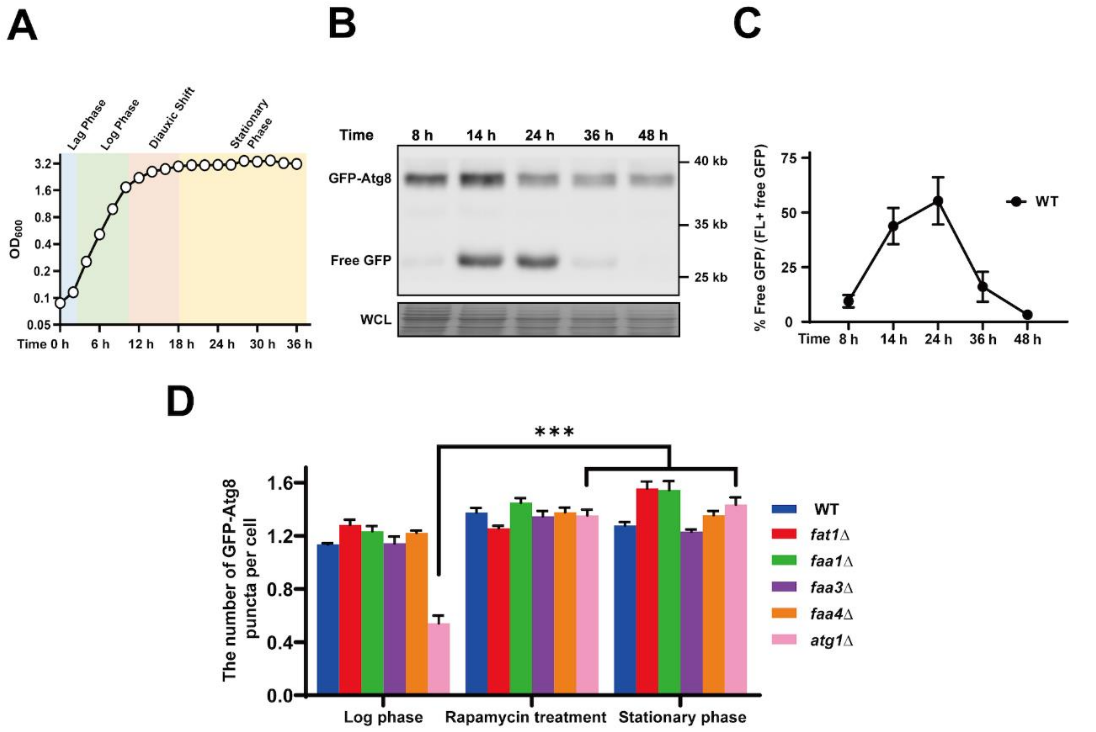

FIG S1 Autophagic flux and growth curve of wild-type cells in SD medium.

(**A**) Growth curve of wild-type strain cultured in SD medium. Cells were precultured in SD medium overnight at 30°C and 210 rpm, then diluted in SD to 0.1 OD_{600} unit/ml, and cultured continuously for 36 h. (**B**) WT cells expressing chromosomal GFP-Atg8 were grown in SD medium for indicated hours. Cells were lysed, and whole-cell lysates (WCL) were subjected to SDS-PAGE and immunoblotting with the anti-GFP antibody or staining with amido black. (**C**) Quantification of the degradation of GFP-Atg8 in (B). The data are means ± SD (n = 4). (**D**) Quantification of the number of GFP-Atg8 puncta in each indicated strain in (Fig. 1A). Data are means ± SD (n = 3). ***, P < 0.001. Scale bar, 5 μm.

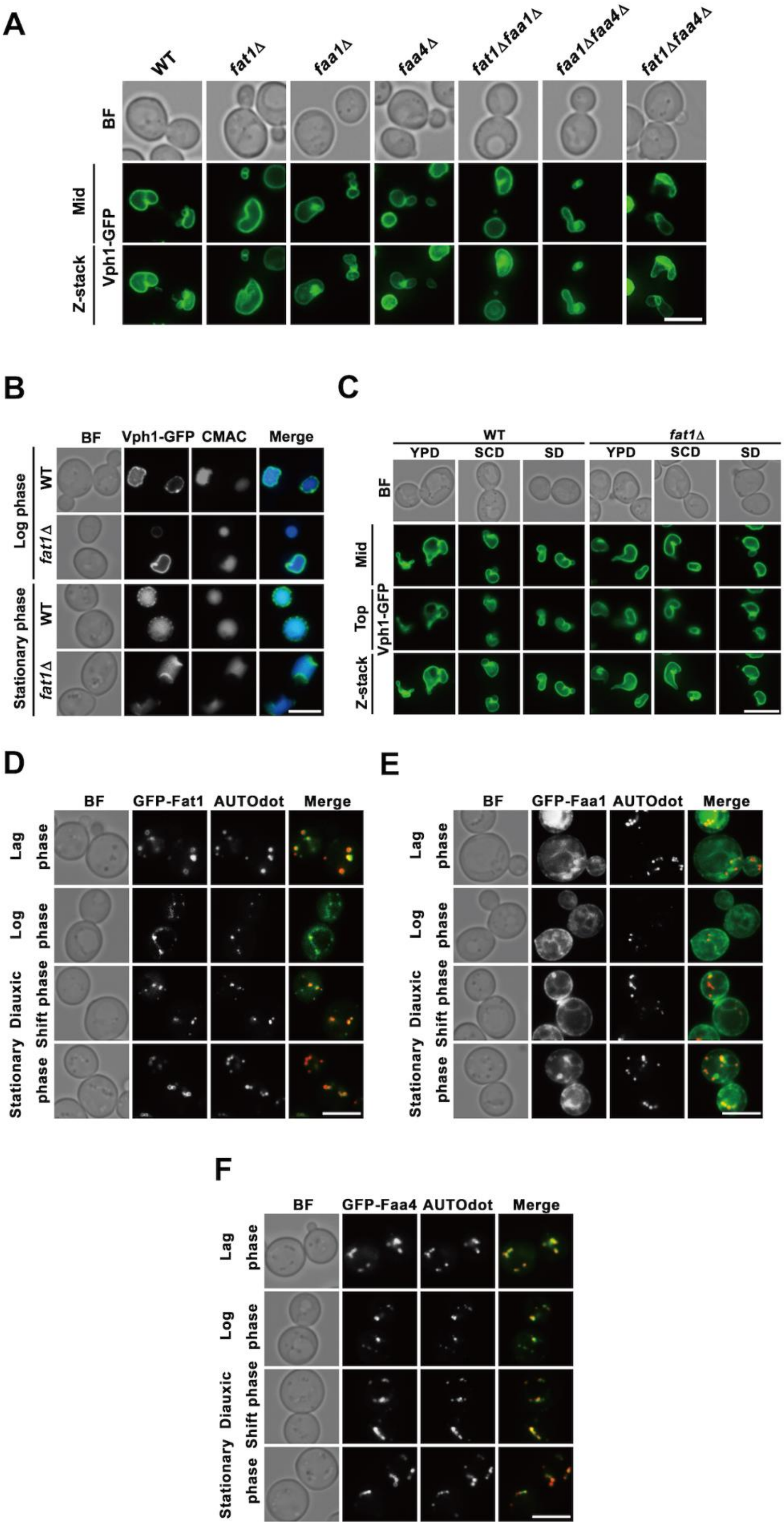

FIG S2 *FAT1* deletion has no effect on log-phase vacuole morphology.

(**A**) Cells of the indicated strains expressing chromosomal Vph1-GFP were grown to log phase in SD medium. (**B**) Vph1-GFP expression strains were stained with CMAC to

visualize vacuolar morphology. (**C**) Vacuole patterns of log-phase WT and *fat1*Δ strains containing Vph1-GFP in YPD, SCD, and SD medium. Top, top image of the vacuole; Mid, middle image of the vacuole. (**D**) to (**F**) The localization of Faa1p, Faa4p and Fat1p in stat-phase yeast cells. Images of cells expressing chromosomal GFP-Fat1 (**D**), GFP-Faa1 (**E**), or GFP-Faa4 (**F**) at various growth phases. To visualize LDs, cells were stained with AUTOdot dye. Top, microscope focus on top tangential area of the vacuole; Mid, focus on the mid-section of the vacuole; Z-stack represents the maximal superimposed projection of vacuole images. Scale bar, 5 μm.

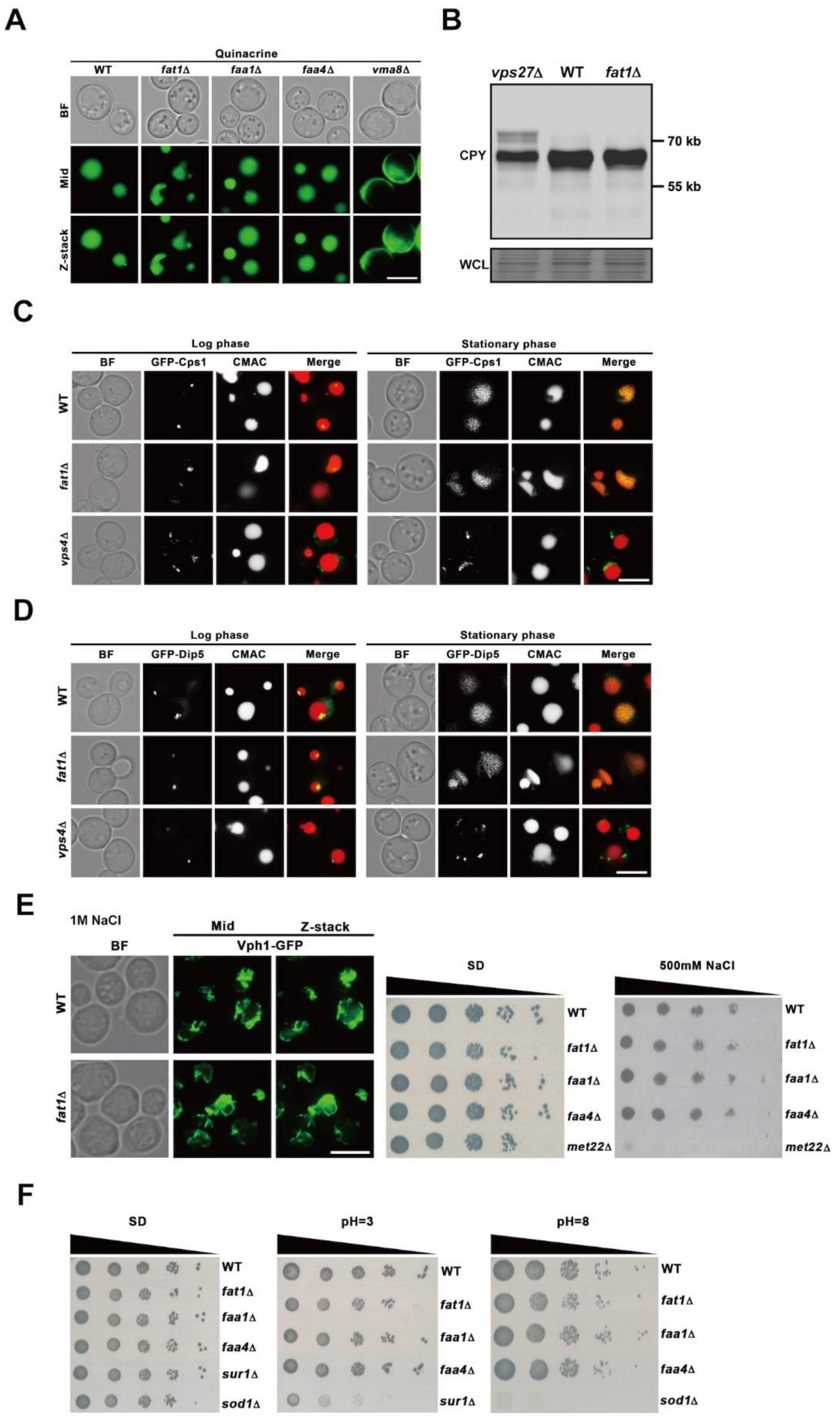

FIG S3 Analysis of basic vacuole functions in *FAT1* deletion strains.

(**A**) Vacuolar acidity was measured. Quinacrine is found in the vacuolar lumen of all tested strains, except for *vma8*Δ. Quinacrine is a fluorescent acidophilic base that diffuses across

cell membranes and accumulates in acidic vacuoles but not in alkaline vacuoles. Vma8p is an essential component of the vacuolar V-type ATPase complex; a *VMA8* mutation results in ineffective H^+ entry into vacuoles and reduced vacuolar acidity. (**B to D**) Analysis of the vacuole sorting along the endolysosomal pathway. (**B**) Evaluation of the CPY pathway. Whole-cell lysates (WCL) from the indicated strains were subjected to SDS-PAGE and immunoblotting with the anti-CPY antibody or staining with amido black. Sorting behavior of *fat1Δ* is similar to WT, as contrasted to the *VPS27* mutant that results in partially immature Carboxypeptidase Y (CPY). The splicing and processing of CPY is blocked, when the CPY pathway from ER synthesis through Golgi processing to ultimate transport to the vacuoles is impaired. Vps27p is a ubiquitin-binding protein that is involved in endosomal protein sorting, and *VPS27* mutation affects CPY splicing and processing. (**C** and **D**) Assessment of the MVB pathway. Sorting of GFP-tagged carboxypeptidase S Cps1p and dicarboxylic acid transporter Dip5p, cargos transported to vacuoles via multivesicular body (MVB) pathway were analyzed in the indicated strains. Vps4p is AAA-ATPase involved in MVB protein sorting, and *VPS4* mutation impairs MVB pathway. (**E** and **F**) The indicated strains were cultured in acidic, alkaline, and hypertonic conditions, as shown. Cells were imaged immediately after being incubated with 1 M NaCl. Cells were spotted in serial 5-fold dilutions and cultured for 2 days at 30°C. Sur1p is a catalytic subunit of mannosylinositol phosphorylceramide synthase, and *SUR1* mutation is sensitive to acidic culture conditions. Sod1p is a cytosolic copper-zinc superoxide dismutase, and *SOD1* mutation is sensitive to alkaline culture conditions. Met22p is a bisphosphate-3'-nucleotidase that is involved in salt tolerance and methionine biosynthesis, and *Met22* mutation is hyperosmotic sensitive. Mid, focus on the mid-section of the vacuole; Z-stack represents the maximal superimposed projection of vacuole images. Scale bar, 5 μm.

TABLE S1. Strains and plasmids used in this study

A. Strains used in this study

Strain Name	Genotype or Description	Ref/Source
W303-1A	*MATa, leu2-3,112 trp1-1 can1-100 ura3-1 ade2-1 his3-11,15*	Thomas and Rothstein, 1989
*fat1*Δ	W303-1A *fat1::clonNat*^{R}	This study
*faa1*Δ	W303-1A *faa1::hyg*^{R}	This study
*faa3*Δ	W303-1A *faa3::hyg*^{R}	This study
*faa4*Δ	W303-1A *faa4::clonNat*^{R}	This study
*atg1*Δ	W303-1A *atg1::hyg*^{R}	This study
*fat1*Δ*faa1*Δ	W303-1A *fat1::clonNat*^{R} *faa1::hyg*^{R}	This study
*faa1*Δ*faa4*Δ	W303-1A *faa1::hyg*^{R} *faa4::clonNat*^{R}	This study
*fat1*Δ*faa4*Δ	W303-1A *fat1::clonNat*^{R} *faa4::hyg*^{R}	This study
GFP-ATG8	W303-1A *atg8::GFP-ATG8 kan*^{R}	This study
GFP-ATG8|*fat1*Δ	GFP-ATG8 containing *fat1::clonNat*^{R}	This study
GFP-ATG8|*faa1*Δ	GFP-ATG8 containing *faa1::hyg*^{R}	This study
GFP-ATG8|*faa3*Δ	GFP-ATG8 containing *faa3::hyg*^{R}	This study
GFP-ATG8|*fat4*Δ	GFP-ATG8 containing *faa4::clonNat*^{R}	This study
GFP-ATG8|*atg1*Δ	GFP-ATG8 containing *atg1::hyg*^{R}	This study
VPH1-GFP	W303-1A *vph1::VPH1-GFP kan*^{R}	This study
VPH1-GFP|*fat1*Δ	VPH1-GFP containing *fat1::clonNat*^{R}	This study
VPH1-GFP|*faa1*Δ	VPH1-GFP containing *faa1::hyg*^{R}	This study
VPH1-GFP|*faa4*Δ	VPH1-GFP containing *faa4::clonNat*^{R}	This study
VPH1-GFP|*fat1*Δ*faa1*Δ	VPH1-GFP containing *fat1::clonNat*^{R} *faa1::hyg*^{R}	This study
VPH1-GFP|*faa1*Δ*faa4*Δ	VPH1-GFP containing *faa1::hyg*^{R} *faa4::clonNat*^{R}	This study
VPH1-GFP|*fat1*Δ*faa4*Δ	VPH1-GFP containing *fat1::clonNat*^{R} *faa4::hyg*^{R}	This study
ERG6-GFP	W303-1A *erg6::ERG6-GFP kan*^{R}	This study
ERG6-GFP|*fat1*Δ	ERG6-GFP containing *fat1::clonNat*^{R}	This study
IVY1-mRuby3	*ivy1::IVY1-mRuby3 clonNat*^{R}	This study
IVY1-mRuby3|*fat1*Δ	IVY1-mRuby3 containing *fat1::clonNat*^{R}	This study
GFP-CPS1	W303-1A *cps1::GFP-CPS1 kan*^{R}	This study
GFP-CPS1|*fat1*Δ	GFP-CPS1 containing *fat1::clonNat*^{R}	This study
GFP-DIP5	W303-1A *dip5::GFP-DIP5 kan*^{R}	This study
GFP-DIP5|*fat1*Δ	GFP-DiP5 containing *fat1::clonNat*^{R}	This study
*vps4*Δ	W303-1A *vps4::hyg*^{R}	This study
GFP-CPS1|*vps4*Δ	GFP-CPS1 containing *vps4::hyg*^{R}	This study
GFP-DIP5|*vps4*Δ	GFP-DiP5 containing *vps4::hyg*^{R}	This study
*vma8*Δ	W303-1A *vma8::clonNat*^{R}	This study
*vps27*Δ	W303-1A *vps27::clonNat*^{R}	This study
*met22*Δ	W303-1A *met22::hyg*^{R}	This study
*sur1*Δ	W303-1A *sur1::hyg*^{R}	This study
*sod1*Δ	W303-1A *sod1::clonNat*^{R}	This study
GFP-FAT1	W303-1A *fat1::GFP-FAT1 kan*^{R}	This study

GFP-FAA1	W303-1A *faa1::GFP-FAA1 kan*^{R}	This study
GFP-FAA4	W303-1A *faa4::GFP-FAA4 kan*^{R}	This study
**B. Plasmids used in this study**		
**Plasmid**	**Description**	**Ref/Source**
PGK1-mRuby3	*pRS316-PGK1-mRuby3 URA3&amp*^{R}	This study
PGK1-Ole1p-mRuby3	*pRS316-PGK1-Ole1p-mRuby3 URA3&amp*^{R} (*PGK1* promoter and terminator)	This study

TABLE S2. Media used in this study

Medium	Components
YPD	Yeast Extract 10 g/L<br>Peptone 20 g/L<br>Glucose 20 g/L<br>For YPDA with extra 20 g/L Agar.
SCD (pH=5.8)	Yeast NitrogenBase w/o Amino Acids (YNB) 6.7 g/L<br>Glucose 20 g/L<br>Adenine 40 mg/L, Arginine 20 mg/L, Aspartic Acid 100 mg/L, Glutamic Acid 100 mg/L, Histidine 20 mg/L, Leucine 60 mg/L, Lysine 30 mg/L, Methionine 20 mg/L, Phenylalanine 50 mg/L, Serine 375 mg/L, Threonine 200 mg/L, Tryptophan 40 mg/L, Tyrosine 30 mg/L, Valine 150 mg/L, and Uracil 20 mg/L<br>For SCDA with extra 20 g/L Agar.
SD (pH=5.8)	YNB 6.7 g/L<br>Glucose 20 g/L<br>Adenine 30 mg/L, Histidine 20 mg/L, Leucine 50 mg/L, Lysine 30 mg/L, Tryptophan 50 mg/L, and Uracil 20 mg/L<br>For SDA with extra 20 g/L Agar.
0.2% SD (pH=5.8)	YNB 6.7 g/L<br>Glucose 2 g/L<br>Adenine 30 mg/L, Histidine 20 mg/L, Leucine 50 mg/L, Lysine 30 mg/L, Tryptophan 50 mg/L, and Uracil 20 mg/L
SD-URA (pH=5.8)	YNB 6.7 g/L<br>Glucose 20 g/L<br>Adenine 30 mg/L, Histidine 20 mg/L, Leucine 50 mg/L, Lysine 30 mg/L, and Tryptophan 50 mg/L<br>For SDA-URA with extra 20 g/L Agar.
OA	YNB 6.7 g/L<br>Adenine 30 mg/L, Histidine 20 mg/L, Leucine 50 mg/L, Lysine 30 mg/L, Tryptophan 50 mg/L, and Uracil 20 mg/L<br>Ethanol 2 mL/L, and Tyloxapol 1 mL/L<br>Oleic acid 1 mL/L

MOVIE S1 View of Vph1-GFP in three dimensions in W303-1A.

MOVIE S2 View of Vph1-GFP in 3D in *fat1*Δ**.**

MOVIE S3 View of Ivy1-mRuby3 localization on the vacuole surface in 3D in W303-1A. CMAC is used to dye the vacuole (pseudo-color as green).

MOVIE S4 View of Ivy1-mRuby3 localization on the vacuole surface in 3D in *fat1*Δ. CMAC is used to dye the vacuole (pseudo-color as green).
